# Temporal and Spatial Distribution Analysis of Atmospheric Pollutants in Chengdu–Chongqing Twin-City Economic Circle

**DOI:** 10.3390/ijerph19074333

**Published:** 2022-04-04

**Authors:** Ning Qi, Xuemei Tan, Tengfei Wu, Qing Tang, Fengshou Ning, Debin Jiang, Tengtun Xu, Hong Wu, Lingxiao Ren, Wei Deng

**Affiliations:** 1 School of Environment and Resources, Chongqing Technology and Business University, Chongqing 400067, China; wonderland892@hotmail.com (X.T.); ningfs_00342@sohu.com (F.N.); jiangdebin@ctbu.edu.cn (D.J.); xutengtun@ctbu.edu.cn (T.X.); wuhong_1985@163.com (H.W.); 2 Institute of Agricultural Resources and Environment, Guangdong Academy of Agricultural Sciences, Guangzhou 510640, China; 3 Chongqing Fushide Environmental Affairs Co., Ltd., Chongqing 401147, China; tangqingtq@163.com; 4 Nanjing Institute of Technology, School of Environmental Engineering, Nanjing 211167, China; rlxjht@njit.edu.cn; 5 Center of Yangtze River Ecological Protection and High Quality Development, Chongqing Academy of Environmental Science, Chongqing 401147, China; dengweidw0821@163.com

**Keywords:** spatial autocorrelation, Moran’s I, air pollution transmission channels, ambient air quality standards, WHO Global Air Quality Guidelines

## Abstract

In order to study the temporal and spatial distribution characteristics of atmospheric pollutants in cities (districts and counties) in the Chengdu–Chongqing Twin-city Economic Circle (CCEC) and to provide a theoretical basis for atmospheric pollution prevention and control, this paper combined Ambient Air Quality Standards (AAQS) and WHO Global Air Quality Guidelines (GAQG) to evaluate atmospheric pollution and used spatial correlation to determine key pollution areas. The results showed that the distribution of atmospheric pollutants in CCEC presents a certain law, which was consistent with the air pollution transmission channels. Except for particulate matter with an aerodynamic diameter equal to or less than 2.5 μm (PM_2.5_) and ozone (O_3_), other pollutants reached Grade II of AAQS in 2020, among which particulate matter with an aerodynamic diameter equal to or less than 10 μm (PM_10_), PM_2.5_, sulfur dioxide (SO_2_), nitrogen dioxide (NO_2_) and carbon monoxide (CO) have improved. Compared with the air quality guidelines given in the GAQG, PM_10_, PM_2.5_, NO_2_ and O_3_ have certain effects on human health. The spatial aggregation of PM_10_ and PM_2.5_ decreased year by year, while the spatial aggregation of O_3_ increased with the change in time, and the distribution of NO_2_ pollution had no obvious aggregation. Comprehensive analysis showed that the pollution problems of particulate matter, NO_2_ and O_3_ in CCEC need to be further controlled.

## 1. Introduction

Currently, atmospheric pollution has become an important environmental problem, which places risk on human health. Many studies show that the release of atmospheric pollutants may cause many adverse health effects such as increased risks of cardiovascular and pulmonary diseases, decreased semen quality, and coronary heart disease [1,2,3]. The rapid developments of transportation and industry cause the discharge of atmospheric pollutants. The emissions of particulate matter with an aerodynamic diameter equal to or less than 2.5 μm (PM_2.5_) and sulfur dioxide (SO_2_) by coal-fired power plants accounted for 6% and 33% of national total emissions in 2010, respectively [4], and the usage of coal accounts for 69% of the total energy consumption [5]. Intense vehicular traffic causes the large emissions of nitrogen dioxide (NO_2_) and carbon dioxide [6]. In order to assess the degree of air pollution, six pollutants such as particulate matter with an aerodynamic diameter equal to or less than 10 μm (PM_10_), PM_2.5_, SO_2_, NO_2_, ozone (O_3_) and carbon monoxide (CO) have been selected to characterize the levels of air pollution. The World Health Organization (WHO) develops the WHO Global Air Quality Guidelines (GAQG) to reduce atmospheric pollutants in order to decrease the enormous health burden resulting from exposure to atmospheric pollution worldwide. In China, the Ambient Air Quality Standard (AAQS) is also set up to protect and improve the living environment and ecological health, and to ensure human health [7].

**Figure 1:**
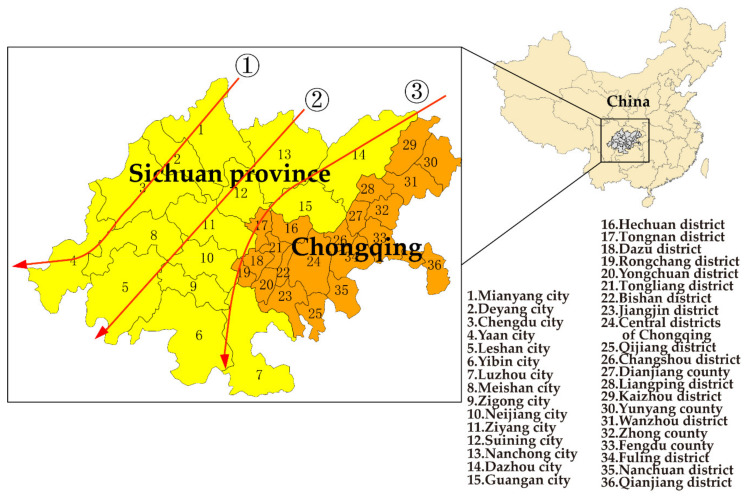
Map of air pollution transmission channels in the Chengdu–Chongqing twin-city Economic Circle (CCEC) [8,9,10].

In January 2020, the construction of the “Chengdu–Chongqing twin-city Economic Circle” (CCEC) was first proposed by the sixth meeting of the Central Finance and Economics Commission, which aims to turn the Chengdu–Chongqing area into an economic circle with its own strengths and distinctive features, as well as a new driver and an important growth engine of the country’s high-quality development. CCEC is made up of some cities in Sichuan province and some districts or counties in Chongqing municipality, which is the urbanization area with the highest development level and the greatest development potential in the western region of China. The high-quality development of CCEC can effectively enhance the economic development and the population-carrying capacity of the urbanization area, which is of great significance to the protection of the ecological environment in the upper reaches of the Yangtze River and of western China. It is also an important part of the implementation of the “Yangtze River Economic Belt” and “the Belt and Road Initiative”. The whole area of the CCEC is 185,000 square kilometers, which includes 29 districts and counties in Chongqing and 15 cities in Sichuan province (Figure 1).

For many years, the atmospheric pollution of CCEC has been particularly serious and complex. Due to the unique topography and climate in Sichuan Basin [11], the atmospheric pollutants accumulate in large quantities and cause the Sichuan Basin to become one of the most heavily polluted areas in China [12,13]. Furthermore, Chongqing is a mountainous city, and its environmental quality is significantly affected by the factors of pollution and dense built environment [14]. The research has shown that there are three air pollution transmission channels in Sichuan Basin due to the effect of the east Asian atmospheric circulation and the Qinghai Tibet Plateau flow field [8,9,10]: (1) Guangyuan → Mianyang → Deyang → Chengdu→ Ya’an; (2) Bazhong → Nanchong→ Suining →Ziyang → Meishan →Leshan; (3) Northern of Chongqing → Dazhou → Guang’an →Nanchong → Suining → Ziyang → Neijiang → Luzhou. Additionally, the pollution of O_3_ has become more and more serious in Sichuan Province since 2015, while the particulate matter has shown characteristics of secondary pollutants [15,16]. The primary pollutants in the atmosphere of Chongqing were PM_10_, nitrogen oxides and SO_2_ in 2007–2014, which showed significant regional differences in air quality [17]. Environmental quality and public health determine the prospect of sustainable development [18]. The prevention and reduction of air pollution has become one of the key issues of current concern for high quality development of the economy in the CCEC.

The aim of this research is to explore the spatiotemporal distribution and pollution degrees of atmospheric pollutants from 2017 to 2020 in the CCEC, and to find the main pollution areas. Based on the results, the reasonable suggestions for pollution control in CCEC were propounded, and the theoretical basis for the coordinated governance of the atmosphere and water environment was provided.

## 2. Data Sources and Methods

### 2.1. Data Sources

In this study, the relevant data of ambient air quality in each area (city in Sichuan province or district and county in Chongqing) of CCEC from 2017 to 2020 were obtained from the ecological and environmental bulletin of Chongqing (http://sthjj.cq.gov.cn/hjzl_249/hjzkgb/, accessed on 31 January 2022) and from nthe ecological and environmental bulletin of each city in Sichuan Province (http://sthjt.sc.gov.cn/sthjt/c104157/hjglnew.shtml, accessed on 31 January 2022). The atmospheric pollutants that were chosen mainly include PM_10_, PM_2.5_, SO_2_, NO_2_, O_3_ and CO.

### 2.2. Evaluation Standards

The evaluation standards for the concentration of atmospheric pollutants were based on the Grade II of AAQS [7,19] (http://www.mee.gov.cn/ywgz/fgbz/bz/bzwb/dqhjbh/dqhjzlbz/201203/t20120302_224165.shtml, accessed on 31 January 2022) and GAQG [20] (https://www.who.int/publications/i/item/9789240034433, accessed on 31 January 2022. The average annual concentrations of PM_10_, PM_2.5_, SO_2_ and NO_2_ were adopted, the 24 h average value of the 95th position for CO was adopted, and the 8 h average value of the maximum daily concentration of the 90th position for O_3_ was adopted (Table 1). The areal interpolation was used to draw the figures of spatial distribution. The indexes in GAQG are stricter than that in AAQS, which is based on the latest evidence of human health caused by air pollution. The purpose of GAQG is to propose new air quality levels, and their interim targets play a role in guiding emission reduction and promoting air quality to reach the level of air quality guidelines. The purpose of the AAQS is based on air quality management. It aims at promoting harmonious and sustainable development between humans and nature [21]; thus, the indexes in AAQS are much closer to the interim target 1 of GAQG.

### 2.3. Methods

Spatial autocorrelation refers to the presence of systematic spatial variation in a mapped variable. The map shows positive spatial autocorrelation where adjacent observations have similar data values. The spatial autocorrelation is often used to detect whether the distribution of variables has spatial dependency, heterogeneity and constitutive properties. Moran’s I is one of the important indexes used to analyze spatial correlation (Equation (1)) [22,23].

(1) $$I=\frac{n\sum_{i=1}^{n}\sum_{j=1}^{n}w_{ij}\left(x_{i}-\bar{x}\right)\left(x_{j}-\bar{x}\right)}{\sum_{i=1}^{n}\sum_{j=1}^{n}w_{ij}\sum_{i=1}^{n}{\left(x_{i}-\bar{x}\right)}^{2}}$$

where n represents the number of the cities and districts; *w_ij_* represents the spatial relationship between region *i* and *j*; *x_i_* and *x_j_*, respectively, are the concentration values of certain atmospheric pollutant in each city; $\bar{x}$ is average concentration value of a certain atmospheric pollutant by study region, $\bar{x}=\frac{1}{n}\sum_{i=1}^{n}x_{i}$. The range of Moran’s I lies between −1 and 1. If the Moran’s I index >0, this implies a positive spatial correlation. Inversely, if the Moran’s I index <0, this indicates a negative correlation [24]. The smaller the value, the stronger the spatial divergence [25].

An alternative approach to measure the relationship typology and intensity are provided by the local indicator of spatial association (LISA) (Equation (2)) [26]. It has four types of distributions, which includes high–high (HH) type, high–low (HL) type, low–high (LH) type and low–low (LL) type. High–high type or low–low type represents spatial clusters of similar high or low concentration values of atmospheric pollutants. Low–high type or high–low type indicates spatial outliers with low concentration values of atmospheric pollutants surrounding high concentration values of atmospheric pollutants or vice versa.

(2) $$L_{i}=\frac{\left(x_{i}-\bar{x}\right)}{S^{2}}\sum_{j=1}^{N}w_{ij}\left(x_{i}-\bar{x}\right)$$

where $S^{2}=\frac{1}{n}\sum_{i=1}^{n}{\left(x_{i}-\bar{x}\right)}^{2};$ S^2^ is the concentration variance of a certain atmospheric pollutant. If *L_i_* > 0, this implies the HH type or LL type. If *L_i_* < 0, this indicates the HL type or LH type [27].

## 3. Result and Discussions

### 3.1. Temporal and Spatial Changes of the Concentrations of Atmospheric Pollutants

#### 3.1.1. PM_10_

**Figure 2:**
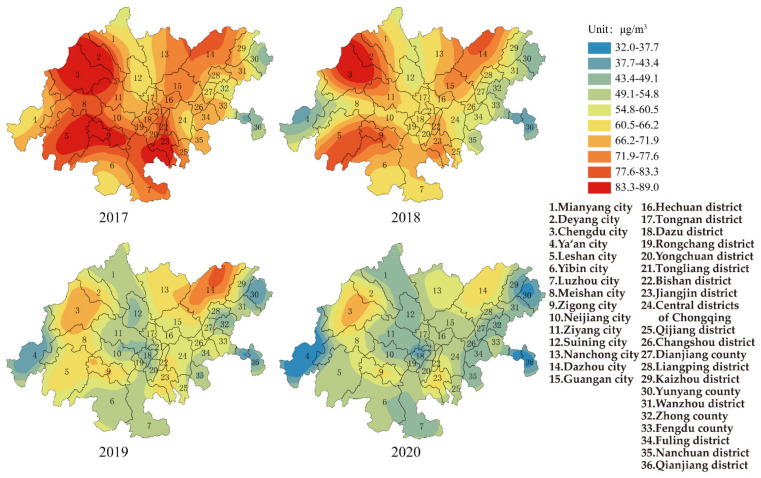
Spatial distribution of PM_10_ in CCEC from 2017 to 2020.

Figure 2 shows the change in PM_10_ for each area in the CCEC during 2017–2020. The average annual concentration of PM_10_ for the whole CCEC was 72.0 μg/m^3^, and the range and standard deviation were 42.0 μg/m^3^ and 9.5 μg/m^3^, respectively, in 2017. The areas with the highest annual average PM_10_ concentration were Zigong city in Sichuan province and Jiangjin district in Chongqing (89 μg/m^3^), and the concentrations were 27.0% higher than Grade II of AAQS. The area with lowest annual average PM_10_ concentration was Qianjiang district in Chongqing (47 μg/m^3^). The number of areas exceeding Grade II of AAQS was about 42.0%. The areas with a higher concentration were mainly located in the cities of Chengdu, Deyang, Leshan, Zigong in Sichuan province and the districts of Jiangjin, Qijiang in Chongqing, and the distribution of PM_10_ was consistent with three air pollution transmission channels. In 2020, the average annual concentration of PM_10_ for the whole CCEC decreased to 50.1 μg/m^3^, while the range and standard deviation were 32.0 μg/m^3^ and 7.2 μg/m^3^, respectively. All cities (districts and counties) met the Grade II of AAQS. The area with the highest concentration of PM_10_ was Chengdu city in Sichuan province (64 μg/m^3^), while the area with the lowest concentration of PM_10_ was Ya’an city in Sichuan province (33 μg/m^3^).

Studies have found that particulate matter in the atmosphere is harmful to human health [28,29]. Based on the interim targets and air quality guideline of PM_10_ in QAGQ, the average annual concentrations of PM_10_ for each area in the CCEC were higher than the air quality guideline (15 μg/m^3^), which caused the potential risk to public health. The numbers of areas exceeding interim target 2 (50 μg/m^3^) and interim target 3 (30 μg/m^3^) were 55.6% and 44.4% respectively. If mortality in a population exposed to PM_10_ at the air quality guideline level was arbitrarily set at 100, then it would be 114 and 106 in populations exposed to PM_10_ at the interim target 2 and 3 levels.

#### 3.1.2. PM_2.5_

**Figure 3:**
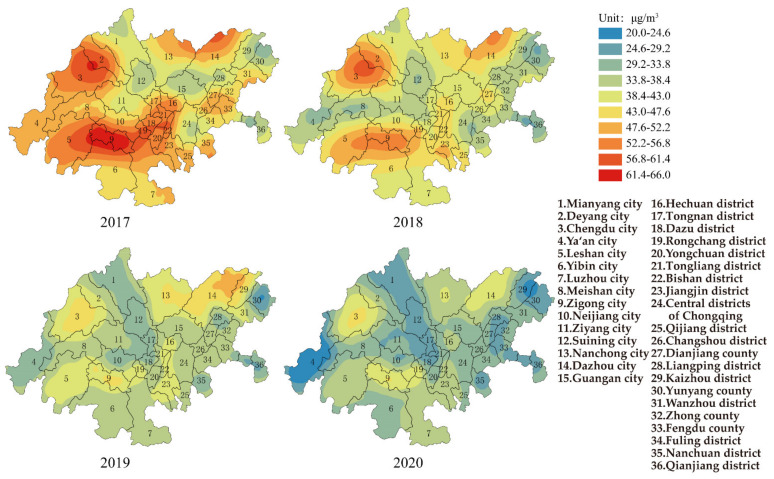
Spatial distribution of PM_2.5_ in CCEC during 2017 to 2020.

Figure 3 shows the change in PM_2.5_ in the CCEC from 2017–2020. The average annual concentration of PM_10_ for the whole CCEC was 48.6 μg/m^3^ (the range was 31.0 μg/m^3^, and standard deviation was 7.2 μg/m^3^) in 2017. In total, 97.0% of the cities failed to reach Grade II of AAQS. The area with the highest concentration was Zigong city in Sichuan province (66 μg/m^3^, which was 89.0% higher than Grade II of AAQS), while the area with the lowest values was Yunyang county in Chongqing (35 μg/m^3^). The heavily polluted areas were mainly located in Chengdu–Deyang and Leshan–Zigong, and the PM_2.5_ distributions were consistent with air pollution transmission channels 1 and 2, which were consistent with the results of Luo et al. [9] and Liao et al. [30]. Some studies showed that air stagnation always happened in the Sichuan–Chongqing region in winter, which caused the high concentration of PM_2.5_ [30]. Furthermore, as one of the most important agricultural regions in China, the combustion of biomass has also caused the severe pollution in Sichuan Basin [31]. The average annual concentration of PM_2.5_ in CCEC decreased to 31.9 μg/m^3^ (the range was 22.9 μg/m^3^, and standard deviation was 5.0 μg/m^3^) in 2020, and 75.0% of the cities met the Grade II of AAQS. The area with highest concentration of PM_2.5_ was Rongchang district in Chongqing (44 μg/m^3^, which was 25.7% higher than the Grade II of AAQS), while the area with lowest value was Ya’an city in Sichuan province (21 μg/m^3^). Based on the mean values, ranges and standard deviations of PM_10_ and PM_2.5_, the control of particulate matter had achieved remarkable improvement in 2020.

It was said by QAGQ that priority should be given to the air quality guidelines of PM_2.5_ when considering the impact of particulate matter. The average annual concentrations of PM_2.5_ for each area in the CCEC were higher than the air quality guidelines (5 μg/m^3^) in 2020. The number of areas exceeding the interim target 1 (35 μg/m^3^), interim target 2 (25 μg/m^3^) and interim target 3 (15 μg/m^3^) were 55.6%, 69.4% and 2.8%, respectively. If mortality in a population exposed to PM_2.5_ at the air quality guideline level was arbitrarily set to 100, then it would be 124, 116 and 108, respectively, in populations exposed to PM_2.5_ at interim target 1, 2 and 3 levels. The results showed that the concentration level of PM_2.5_ causes a high risk to public health and should be given priority to control.

It was found that the higher the ratio value of PM_2.5_/PM_10_, the heavier the influence on the atmospheric environment [32]. The mean value of the PM_2.5_/PM_10_ ratio in Beijing was 0.72, which ranged from 0.31~0.96 [32]. The PM_2.5_/PM_10_ ratio values of Kaohsiung and Hong Kong were around 0.62 and 0.63 [33,34], respectively. The ratio values of PM_2.5_/PM_10_ in the whole CCEC were 0.68, 0.66, 0.66 and 0.64 from 2017 to 2020, respectively. It was shown that the larger the proportion of PM_2.5_ in particulate matter, the higher the risk to public health. The range of PM_2.5_/PM_10_ ratio in cities of Sichuan province in the CCEC were between 0.63–0.64. The ratio of PM_2.5_/PM_10_ showed a small decrease from 0.7 to 0.64 in the districts (counties) of Chongqing in the CCEC. The results showed that the pollution of particulate matter in Chongqing was much heavier than that in Sichuan province.

#### 3.1.3. O_3_

**Figure 4:**
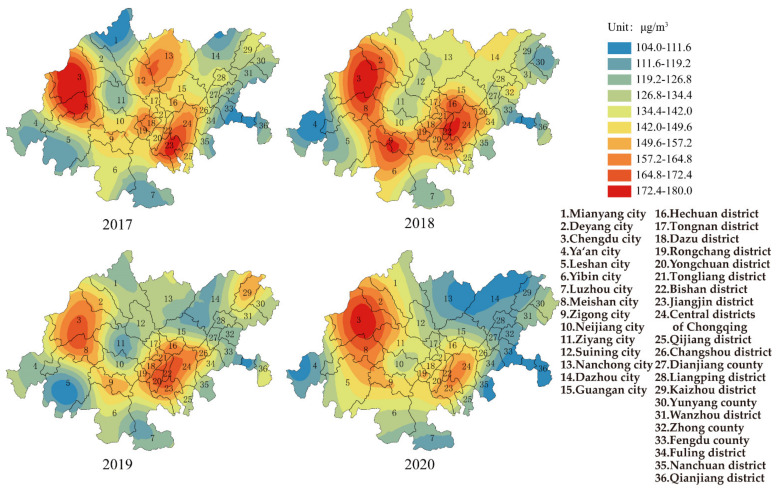
Spatial distribution of O_3_ in CCEC during 2017 to 2020.

Figure 4 shows the change in O_3_ in the CCEC from 2017–2020. In 2017, the concentration of O_3_ for the whole CCEC was 139.6 μg/m^3^ (the range was 60.0 μg/m^3^, and standard deviation was 15.6 μg/m^3^), and 11.0% of the areas failed to reach Grade II of AAQS. The area with the highest concentration of O_3_ was the Jiangjing district in Chongqing (164 μg/m^3^), which was 9.0% higher than Grade II of AAQS, and the area with the lowest concentration of O_3_ was Mianyang city in Sichuan province (114 μg/m^3^). The mean values of O_3_ were slightly increased at 144.8 μg/m^3^ in 2018 and 140.6 μg/m^3^ in 2019. The distributions mainly located in Chengdu–Meishan, Chongqing, and Zigong were consistent with three air pollution transmission channels. Based on our results, the pollution of O_3_ was severe in the area of Chengdu. Yang et al. found that the gasoline vehicle exhaust and the use of solvents was the main reason [35]. Due to the low atmospheric pressure, small pressure gradient, and the stable weather, the condition of horizontal diffusion was bad for the diffusion of pollutants [36]. The photochemical reactions were promoted in Chongqing due to the increase in oxidation after 2016, and the exogenous input of VOCs in nearby areas led to the increase in O_3_ concentration [37]. The concentration of O_3_ for the whole CCEC decreased to 133.8 μg/m^3^ (the range was 65.0 μg/m^3^, and standard deviation was 16.4 μg/m^3^) in 2020. The area with the highest concentration of O_3_ was Chengdu city in Sichuan province (169 μg/m^3^), which was 6.0% higher than Grade II of AAQS, and the area with the lowest concentration of O_3_ was Qianjiang district in Chongqing (104 μg/m^3^). In total, 97.2% of the areas met the Grade II of AAQS.

Long-term exposure to high concentrations of O_3_ could cause chronic damage to the human body. The concentrations of O_3_ for each area in the CCEC were higher than the air quality guidelines (100 μg/m^3^). The number of areas exceeding interim target 1 (160 μg/m^3^) and interim target 2 (120 μg/m^3^) were 2.8% and 72.2%, respectively. If mortality in a population exposed to ozone at the air quality guideline level was arbitrarily set at 100, then it would be 103 and 101 in the populations exposed to ozone at the interim target 1 and 2 levels. The results suggest that more strict measures are needed to be implemented for O_3_ control in the future.

#### 3.1.4. SO_2_

**Figure 5:**
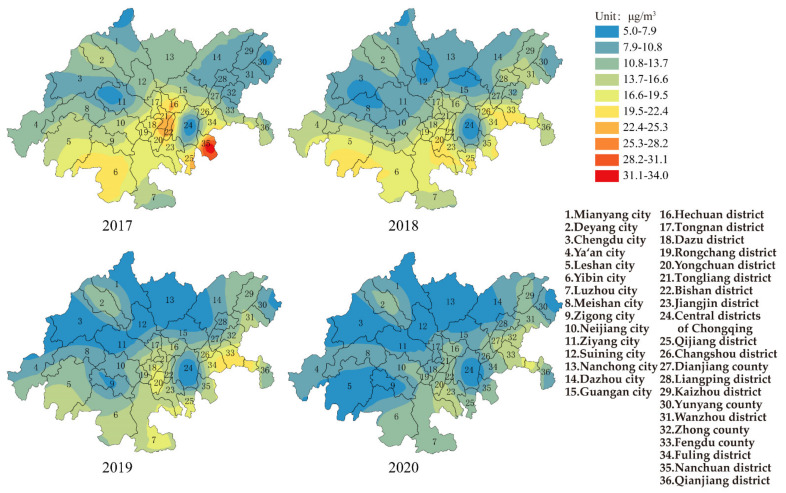
Spatial distribution of SO_2_ in CCEC during 2017 to 2020.

The average annual concentration of SO_2_ for the whole CCEC met Grade II of AAQS in 2017 (Figure 5). The mean value, range, and standard deviation were 15.4 μg/m^3^, 26.0 μg/m^3^, and 5.9 μg/m^3^, respectively. The area with the highest annual average SO_2_ concentration was Nanchuan district in Chongqing (34 μg/m^3^), while the areas with the lowest annual average SO_2_ concentration were the counties of Yunyang and Zhong in Chongqing (8 μg/m^3^). The average annual concentration of SO_2_ for the whole CCEC decreased to 10.3μg/m^3^ in 2020; the range and standard deviation were 10.0 μg/m^3^ and 2.8 μg/m^3^, respectively. In recent years, the average annual concentration of SO_2_ was far below Grade II of AAQS. The Sichuan Basin has suffered from pollution of SO_2_ for the past decades, because 80% SO_2_ concentrations in some areas of the Sichuan Basin was from industries such as chemical, textile, electronics, etc. [38]. Since the implementation of “the Division Plan of Acid rain and Sulfur dioxide Pollution Control Areas”, the control of SO_2_ achieved obvious improvement, basically removing the long-term problems of acid rain and sulfur dioxide pollution. The mean concentration of SO_2_ in areas of Sichuan province in CCEC were reduced from 13.3 μg/m^3^ in 2017 to 8.2 μg/m^3^ in 2020, and the mean concentration of SO_2_ in areas of Chongqing in the CCEC were reduced from 17.0 μg/m^3^ in 2017 to 12.2 μg/m^3^ in 2020. The concentration of SO_2_ in the air depends on the consumption of coal [39]. The proportion of coal consumption in Sichuan province was 32.0% in 2019, while that in Chongqing was 53.0%. Therefore, the control of SO_2_ in Sichuan province was better.

#### 3.1.5. NO_2_

**Figure 6:**
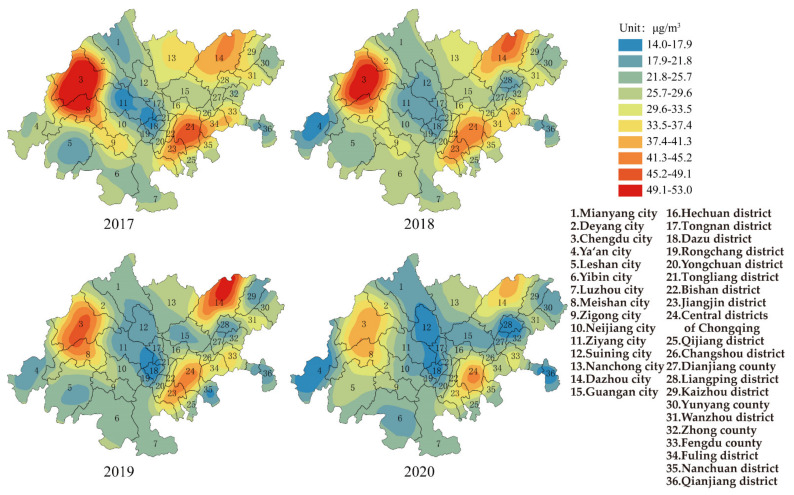
Spatial distribution of NO_2_ in CCEC from 2017 to 2020.

In 2017, the average annual concentration of NO_2_ for the whole CCEC was 29.9 μg/m^3^, the range and standard deviation were 35.0 μg/m^3^ and 7.5 μg/m^3^, respectively (Figure 6). The number of areas exceeding Grade II of AAQS was about 11.0%. The area with the highest and lowest concentrations of NO_2_ were Chengdu city in Sichuan province (53 μg/m^3^) and Dazu district in Chongqing (18 μg/m^3^). The distributions of NO_2_ were consistent with air pollution transmission channels 1 and 3, which were mainly located in the cities of Chengdu, Meishan, Dazhou, the central districts and Jiangjin district of Chongqing. The rapid development of urbanization in these areas has led to a gradual increase in vehicles, and one of the main sources of NO_2_ was traffic emissions [1]. The average annual concentration of NO_2_ for the whole CCEC decreased to 24.1 μg/m^3^ in 2020, and the range and standard deviation were 24.0 μg/m^3^ and 5.9μg/m^3^, respectively. Each area in the CCEC reached Grade II of AAQS, which meant that the control measures of NO_2_ in the CCEC also had certain effects in the past years.

The average annual concentrations of NO_2_ for each area in the CCEC were lower than interim target 1 of QAGQ (40 μg/m^3^) in 2020. The number of areas exceeding interim target 2 (30 μg/m^3^), interim target 3 (20 μg/m^3^), and the air quality guidelines (10 μg/m^3^) were 11.1%, 58.3%, and 27.7%, respectively. If all-cause mortality in a population exposed to nitrogen dioxide at the AQG level was arbitrarily set at 100, then it would be 104 and 102 in populations exposed to nitrogen dioxide at the interim target 2 and 3 levels. The results showed that the potential risk to public health caused by NO_2_ pollution might still exist.

#### 3.1.6. CO

**Figure 7:**
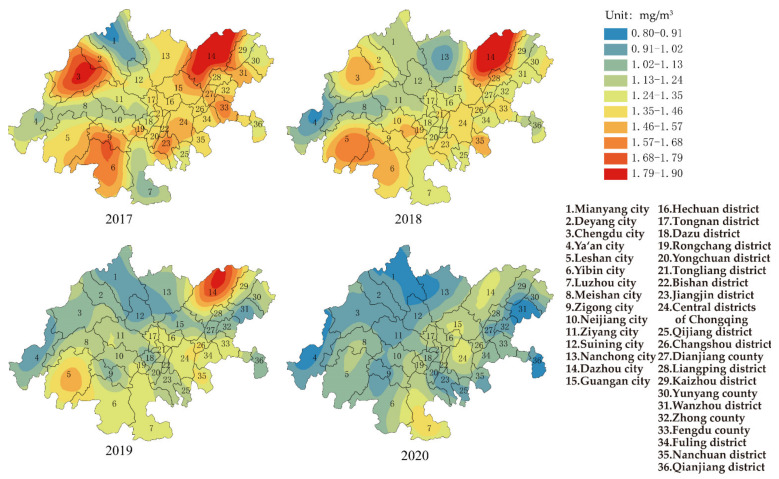
Spatial distribution of CO in CCEC during 2017 to 2020.

Figure 7 shows the change in CO in the CCEC from 2017–2020. The results showed that the control of CO in the CCEC had always been effective, since the concentration of CO for the whole CCEC was 1.4 mg/m^3^ (the range and standard deviation were 0.9 mg/m^3^ and 0.2 mg/m^3^, respectively). The concentration of CO was far below Grade II of AAQS and the air quality guideline in QAGQ, which meant no potential risk to public health. The area with the highest and lowest concentrations of CO was Dazhou city (1.9 mg/m^3^) and Mianyang city (1.0 mg/m^3^) in Sichuan province, respectively. In 2020, the concentration of CO for the whole CCEC slightly decreased to 1.1 mg/m^3^; the range and standard deviation were 0.5 mg/m^3^ and 0.1 mg/m^3^, respectively.

In summary, the controls of SO_2_ and CO in CCEC were effective. The pollutions of PM_10_, PM_2.5_, and NO_2_ had obvious improvement, while the control of O_3_ was not obvious. The concentrations of PM_10_, PM_2.5_, O_3_, and NO_2_ in 2020 were still higher than the air quality guidelines in QAGQ, which meant that the potential risk to public health still exited. The terrain of CCEC was quite complex; the basin in Sichuan province and mountains in Chongqing caused the accumulation of atmospheric pollutants. The pollutions of PM_10_, PM_2.5_, and NO_2_ were quite severe in 2017. Furthermore, the distributions of PM_10_, PM_2.5_, O_3_ and NO_2_ were consistent with three air pollution transmission channels, which verified the unique geographical and climatic factors influencing the distributions. Since the revised version of “The Environmental Protection Law of People’s Republic of China” came into force in 2015, the concentrations of PM_2.5_ and SO_2_ have decreased over time [40]. There were 27 key tasks that had been completed to improve air quality, meet the capacity of atmospheric environment, and control the pollution of PM_2.5_ and nitrogen oxide in Chongqing city since 2018. The air quality in Sichuan province was also improved by dividing the key areas of air pollution prevention and control, carrying out stricter environmental protection standards, and implementing environmental monitoring systems since 2019. Meanwhile, the interventions to control COVID-19 might improve air quality. Studies have found reductions in NO_2_ and PM_2.5_ concentrations during the pandemic [41,42]. The air quality of China was also significantly improved due to the anti-epidemic measures [43,44]. The reduction in human activities (traffic and industry) led to the decrease in atmospheric pollutants [45]. With the economic activities resumed, the effect of improvements on air quality will be offset in the short term. Some sustainable policies must be carried out to tackle air pollution in the post-pandemic era [44]. However, the heavily polluted areas still existed in 2020. Thus, the heavily polluted areas caused by PM_10_, PM_2.5_, O_3_, and NO_2_ were analyzed by spatial autocorrelation.

### 3.2. Spatial Autocorrelation of Air Pollution in CCEC

#### 3.2.1. Global Spatial Autocorrelation

Table 2 shows the Global Moran′s I of PM_10_, PM_2.5_, NO_2_, and O_3_ during 2017–2020. The Global Moran’s I values of PM_10_ and PM_2.5_ were 0.49 and 0.35 (*p* < 0.05, Z > 1.65) in 2017, respectively. The certain aggregation characteristics of PM_10_ and PM_2.5_ with positive spatial correlation were shown in the CCEC, and there was an obvious tendency of aggregation in heavily polluted areas. The Global Moran′s I values of PM_10_ and PM_2.5_ decreased to 0.21 and 0.06 in 2020, which meant that the spatial aggregations of PM_10_ and PM_2.5_ were changed from aggregation distribution to random distribution, due to the implementation of atmospheric control measures. The Global Moran′s I value of O_3_ increased from 0.21 in 2017 to 0.57 in 2020, and the spatial aggregation became significant. The Global Moran′s I value of NO_2_ did not show any significance, which meant that there was no significant spatial aggregation of NO_2_ in CCEC.

#### 3.2.2. Local Spatial Autocorrelation

**Figure 8:**
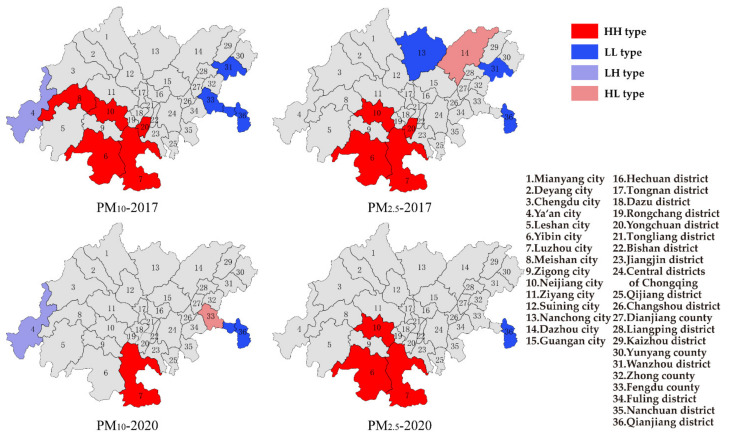
LISA cluster of PM_10_ and PM_2.5_ in 2017 and 2020.

The cities of Yibin, Neijiang, Luzhou, Meisan in Sichuan province and Yongchuan district in Chongqing showed the HH type of PM_10_ in 2017 (Figure 8), which meant that these areas suffered from heavy pollution of PM_10_. The areas of Wanzhou district, Fengdu county, and Qianjiang district in Chongqing showed the LL type of PM_10_ in 2017, while the Ya’an city in Sichuan province showed the LH type. Meanwhile, the cities of Yibin, Neijiang, Luzhou in Sichuan province and the Yongchuan district in Chongqing showed the HH type of PM_2.5_ in 2017. Nanchong city in Sichuan province and the districts of Wanzhou, Qianjiang in Chongqing showed the LL type of PM_2.5_, while Dazhou city in Sichuan province showed the HL type. The results showed that the distribution of particulate matter had obvious regional aggregation; the heavily polluted areas were consistent with the distribution of air pollution transmission channels, which were at the end of the channels. The cities of Zigong, Yibin, Neijiang and Luzhou were traditional industrial bases, and the large-scale and intensification of heavy polluting industries also resulted in the aggregation of heavily polluted areas [27]. In 2020, with the implementation of atmospheric control measures, the number of cities with the HH type of PM_10_ decreased obviously, and the spatial aggregation became weak. Meanwhile, the number of cities with the HH type of PM_2.5_ was barely changed. Based on the results, the cities with the HH type of PM_10_ and PM_2.5_ located at the end of the three transmission channels and the control of PM_10_ were better than the control of PM_2.5_. The southern part of the CCEC still deserved key attention in the future control of particulate matter.

**Figure 9:**
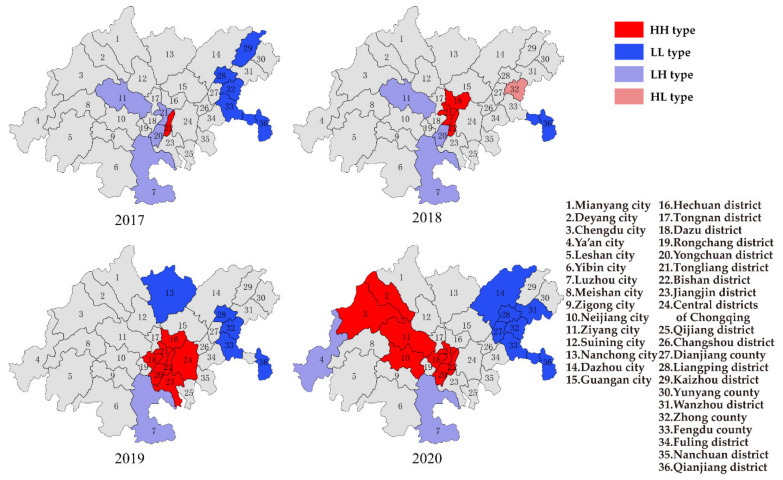
LISA cluster of O_3_ from 2017 to 2020.

The areas of Wanzhou district, Liangpin district, Zhong county, and Fengdu county in Chongqing showed the LL type of O_3_ in 2017 (Figure 9). These areas had relatively lighter O_3_ pollution. Few cities showed the HH type of O_3_. Meanwhile, the LH type of O_3_ was shown in the cities of Ziyang, Luzhou in Sichuan province and the districts of Tongliang, Yongchuan in Chongqing. The distribution of O_3_ in the CCEC was still random in 2017. The HH type of O_3_ became more and more obvious year by year, which was mainly located in the cities of Chengdu, Deyang, Neijiang, Ziyang in Sichuan province and districts of Bisan, Tongliang, Yongchuan, and Dazu in Chongqing, which was consistent with the middle reach of the three air pollution transmission channels. Based on the distributions of particulate matter and O_3_, the degree of pollution in Sichuan province was heavier, due to the low topography of the Sichuan Basin, which was not conducive to the discharge of pollutants. Furthermore, the relatively developed economy, large population density and high industrial density in Sichuan province caused the high emission of pollutants [13]. Therefore, the areas with a high concentration of O_3_ should be controlled to prevent the expansion of heavy polluted areas.

**Figure 10:**
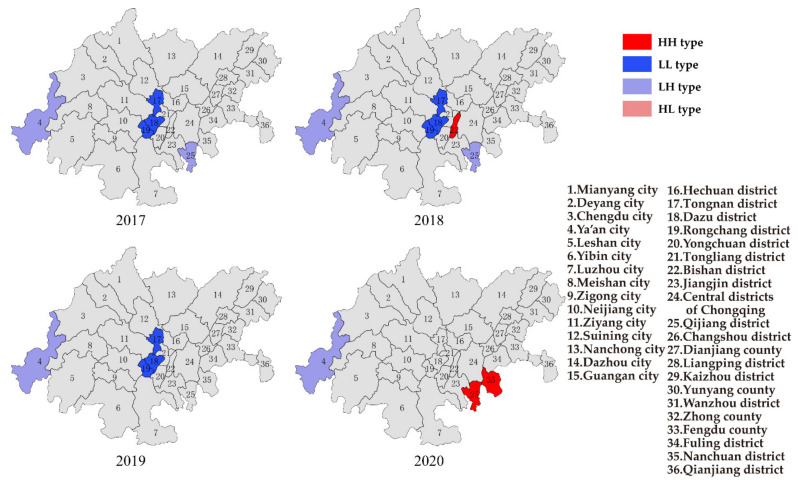
LISA cluster of NO_2_ from 2017 to 2020.

In 2017, the districts of Tongnan, Dazu, and Rongchang in Chongqing showed the LL type of NO_2_, and the city of Ya’an in Sichuan province and district of Qijiang in Chongqing showed the LH type (Figure 10). In 2020, the distribution of NO_2_ in CCEC was still random, yet the districts of Nanchuan and Qijiang in Chongqing showed the HH type. Based on the concentration of NO_2_ in 2020, the average annual concentration of NO_2_ for the whole CCEC was 24.1 μg/m^3^, while the average annual concentration of NO_2_ in the districts of Nanchuan and Qijiang in Chongqing was 25.5 μg/m^3^. Since the obvious effect was achieved on NO_2_ control in the CCEC, the average annual concentration of NO_2_ decreased, and the high concentration of NO_2_ in the districts of Nanchuan and Qijiang in Chongqing caused these areas to be the HH type. Therefore, the control of NO_2_ in the districts of Nanchuan and Qijiang in Chongqing should be further strengthened in the future.

## 4. Conclusions

We have investigated the temporal and spatial distribution of atmospheric pollutants in the CCEC from 2017 to 2020. The concentrations of PM_10_, SO_2_, NO_2_, and CO met the Grade II of AAQS in 2020, due to the implementation of atmospheric control measures. The average annual concentration of SO_2_ for the whole CCEC decreased from 15.4 μg/m^3^ in 2017 to 10.3 μg/m^3^ in 2020, and the long-term problems of acid rain and sulfur dioxide pollution were basically eliminated. The concentrations of PM_10_ and PM_2.5_ also improved significantly; there were 30.4% and 34.4% reductions for the average annual concentration of PM_10_ and PM_2.5_. The concentration change of O_3_ was not obvious, yet 97.2% of the areas met the Grade II of AAQS in 2020. The distributions of PM_10_, PM_2.5_, O_3_, and NO_2_ were consistent with three air pollution transmission channels, which meant that the distribution of atmospheric pollutants was influenced by topographic and climatic conditions.

The concentrations of PM_10_, PM_2.5_, O_3_, and NO_2_ were far beyond the air quality guidelines in QAGQ in 2020. The purpose of the air quality guidelines was to illustrate the minimum impact of atmospheric pollutants on human health. The concentration levels of PM_10_, PM_2.5_, O_3_, and NO_2_ still had certain impacts on human health, and it is necessary to reduce the concentration of these atmospheric pollutants by using interim targets in QAGQ.

Based on the results of spatial autocorrelation of air pollution in the CCEC, the spatial aggregation of PM_10_ was significantly reduced, and the number of areas with the HH type of PM_10_ decreased in 2020. Meanwhile, the HH type of PM_2.5_ was mainly located in the southern part of CCEC, and it barely changed in 2020. The spatial aggregation of O_3_ became obvious in 2020, and the HH type of O_3_ was shown in the central and northwest parts of the CCEC. The spatial aggregation of NO_2_ was random during 2017–2020, yet the districts of Nanchuan and Qijiang in Chongqing showed the HH type of NO_2_.

In summary, the key control areas of particulate matter should focus on the southern part of the CCEC, and the control of industrial pollution sources in the cities of Zigong, Yibin, Neijiang and Luzhou in Sichuan province should be strengthened. It was suggested that the growth rate of coal-fired power plants should be controlled strictly, the proportion of coal and gas in electricity needs to be optimized, and the transformation of the steel industry to achieve ultra-low emissions should be accelerated. The key control areas of O_3_ should focus on the central and northwest parts of the CCEC. It was recommended to reduce the emission of NOx and VOCs in these regions, especially focusing on the sources of scattered polluted enterprises and key industry VOCs emissions. The distribution of NO_2_ pollution was random to some extent, yet NO_2_ pollution in the southern part of the CCEC is still worth paying attention to.

## Figures and Tables

**Table 1:** Limit values of atmospheric pollutants in AAQS and GAQG [7,19,20].

Atmospheric Pollutants	Ambient Air Quality	WHO Global Air Quality Guidelines					
	The Secondary Limits	Interim Target 1	Interim Target 2	Interim Target 3	Interim Target 4	Air Quality Guideline	Unit
PM_10_	70	70	50	30	20	15	μg/m^3^
PM_2.5_	35	35	25	15	10	5	μg/m^3^
SO_2_ *	60	-	-	-	-	-	μg/m^3^
O_3_	160	160	120	-	-	100	μg/m^3^
NO_2_	40	40	30	20	-	10	μg/m^3^
CO	4	7	-	-	-	4	mg/m^3^

* No average annual concentration of SO_2_ was given in GAQG.

**Table 2:** Global Moran′s I of atmospheric pollutants.

Year	PM_10_	PM_2.5_	NO_2_	O_3_
2017	0.49	0.35	0.14	0.21
2018	0.43	0.31	0.08	0.33
2019	0.17	0.05	0.04	0.29
2020	0.21	0.06	0.10	0.57

## Data Availability

The data that support the findings of this study are available from the corresponding author upon reasonable request.
